# Editorial: The development and clinical application of innovative optical ophthalmic imaging techniques

**DOI:** 10.3389/fmed.2022.1058069

**Published:** 2022-11-15

**Authors:** Jinze Zhang, Claude Boccara, Kate Grieve, Yali Jia, Peng Xiao

**Affiliations:** ^1^State Key Laboratory of Ophthalmology, Zhongshan Ophthalmic Center, Sun Yat-sen University, Guangzhou, China; ^2^Institut Langevin, ESPCI Paris, CNRS, PSL University, Paris, France; ^3^Paris Eye Imaging Group, Vision Institute, Quinze Vingts National Ophthalmology Hospital, INSERM, CNRS, Paris, France; ^4^Casey Eye Institute, Oregon Health and Science University, Portland, OR, United States

**Keywords:** ophthalmic imaging, ophthalmic image analysis, artificial intelligence, optical imaging, ophthalmic optical coherence tomography (OCT) equipment

According to the report of the World Health Organization, ophthalmic diseases are the third major group of diseases that severely affect people's quality of life (1). As the most important sensory organization, the eye plays an irreplaceable role in obtaining external information and performing effective communication with its sophisticated optical refractive system and microvascular system. Precise structural and functional evaluation and analysis of the eye based on optical imaging modalities is important for the early and accurate diagnosis and therapeutic evaluation of ophthalmic diseases. At the same time, as the only tissue in the body where blood vessels can be directly observed *in vivo*, many systemic diseases can also be detected or diagnosed through ocular imaging. With the breakthrough of cutting-edge optical imaging and artificial intelligence technologies, high-resolution, multi-dimensional, and intelligent have become the key development trends of ophthalmic imaging methods, with their core technical features of functional imaging, multimodal information fusion, and artificial intelligence (AI) assisted analysis (2).

In fact, translational research in novel optical imaging modalities, such as optical coherence tomography (OCT) and confocal microscopy (3, 4), has greatly advanced the precise management of ocular and related diseases by facilitating ophthalmic imaging with more comprehensive physiological and pathological information due to their high-resolution or 3-dimensional (3D) imaging properties. In the meantime, new-generation optical imaging systems with specific technical features continue to emerge: non-contact full-field OCT provides cellular-resolution images of both anterior and posterior eye (5, 6); combining traditional optical imaging systems with adaptive optics (AO) enhances the resolution by removing the limits imposed by intrinsic eye aberrations (7); OCT angiography based on OCT or Doppler imaging techniques offer the possibility of assessing vascular structural network and blood flow function (8–10); AI-assisted systems enable objective image assessment and automated disease diagnosis and large-scale screening (11, 12). Only with the development of these innovative ophthalmic imaging instruments and techniques, can we continue to advance our understanding, diagnosis, and treatment of ocular diseases.

In this Research Topic, 13 original research articles report their development or clinical verification of new ophthalmic imaging equipment or image analysis algorithms. Nine studies involved the evaluation and application of new optical ophthalmic imaging devices. Wang et al. developed and applied a visible light optical coherence tomography device to healthy eyes and determined the microvascular oxygen saturation baseline in the paracentral macular sulcus. Chen H. et al. compared the segmentation error rate of anterior chamber volume and iris volume measured automatically using anterior segment OCT in both narrow-angle and wide-angle eyes. Using fluorescence adaptive optics scanning laser ophthalmoscopy (SLO), Vienola et al. demonstrated for the first time the cellular level structural changes of the retinal pigment epithelial cell mosaic in Torpedo Maculopathy. Lu et al. evaluated the central and peripheral refraction measurement reproducibility of multispectral optometry and assessed their agreement in myopic patients with subjective refraction measurement. Chen L. et al. evaluated the performance of magnetic resonance imaging based radiomics in the differential diagnosis of orbital cavernous hemangioma and orbital nerve sheath tumor. Xin et al. found a decrease in lens thickness, outer retinal layer thickness and cone photoreceptor density in myopia subjects using swept-source OCT and AO assisted fundus photography. Fan et al. evaluated the effectiveness of a new-generation portable 3D imaging device to assess the anthropometry periocular region. Combining both SLO and OCT, Paques et al. documented the temporal changes over months of the cell-scale dynamics in lesion borders during the progression of geographic atrophy. The study by Cai et al. thoroughly characterized and quantified the human retinal axial motion using 200 kHz spectral-domain OCT (SD-OCT) with high axial resolution. Four studies proposed novel image analysis methods for ophthalmic evaluation. Xu L. et al. showed a decrease in stereopsis with magnification increase in aniseikonia using a phoropter and two 4K smartphones incorporated with contour-based and random point-based stereograms. Xu J. et al. developed a new retinal blood vessel segmentation algorithm for limited image data and gold-standard annotations based on so-called few-shot learning to assist laser surgery for central serous chorioretinopathy. To resolve the chromatic dispersion problem in OCT imaging, which was typically addressed with hardware methods, Ahmed et al. developed an numerical method with deep learning network for robust automatic dispersion compensation in OCT. Pfäffle et al. demonstrated that conducting appropriate signal post-processing in full-field swept-source OCT can provide good phase stability and spatial resolution for retinal neuronal signal processing analysis, opening up the possibility of using phase-sensitive FF-SS-OCT for functional evaluation of different retinal cell types *in vivo*.

Besides, four reviews or meta-analysis articles discuss the development and application of new ophthalmic imaging techniques. Tan et al. conducted a meta-analysis on the application of optical coherence tomography angiography in systemic hypertension. Kim et al. summarize recent advances of functional OCT for intrinsic optical signal (IOS) imaging and computational IOS processing based on OCT intensity or phase analysis. Jiang and Qi evaluated the diagnostic sensitivity and specificity of SD-OCT for polypoidal choroidal vasculopathy by conducting a meta-analysis study. Alexopoulos et al. thoroughly summarized the recent advances in various optical ophthalmic imaging techniques, discussed their potential contributions for daily clinical settings, and also evaluated the important contribution of artificial intelligence in the field of ophthalmic imaging.

In summary, this Research Topic focuses on the development of a new generation of ophthalmic optical imaging equipment and intelligent quantitative image analysis techniques; their clinical application for more accurate and effective imaging diagnosis and pathogenesis of ophthalmic related diseases; and finally provides insights into the latest translational optical ophthalmic imaging research and its applications in resolving clinical needs.

## Author contributions

All authors listed have made a substantial, direct, and intellectual contribution to the work and approved it for publication.

## Funding

This work is supported by the National Natural Science Foundation of China (No. 81901788), Guangdong Basic and Applied Basic Research Foundation (No. 2022A1515011486), and Guangzhou Science and Technology Program (No. 202002030412).

## Conflict of interest

The authors declare that the research was conducted in the absence of any commercial or financial relationships that could be construed as a potential conflict of interest.

## Publisher's note

All claims expressed in this article are solely those of the authors and do not necessarily represent those of their affiliated organizations, or those of the publisher, the editors and the reviewers. Any product that may be evaluated in this article, or claim that may be made by its manufacturer, is not guaranteed or endorsed by the publisher.
